# Non-synonymous genetic variation in exonic regions of canine Toll-like receptors

**DOI:** 10.1186/2052-6687-1-11

**Published:** 2014-10-22

**Authors:** Anna Cuscó, Armand Sánchez, Laura Altet, Lluís Ferrer, Olga Francino

**Affiliations:** Molecular Genetics Veterinary Service. Veterinary School, Universitat Autònoma de Barcelona, Barcelona, Spain; Vetgenomics. Ed Eureka. Parc de Recerca UAB, Barcelona, Spain; Department of Clinical Sciences, Cummings School of Veterinary Medicine, Tufts University, North Grafton, MA USA

**Keywords:** TLRs, Toll-like receptor, Polymorphism, SNPs, Non-synonymous SNPs, Canine, Dog, Innate immunity

## Abstract

**Background:**

Toll-like receptors (TLRs) are pattern recognition receptors (PRRs) considered to be the primary sensors of pathogens in innate immunity. Genetic variants could be associated to differences in breed innate immune response to pathogens and thus to susceptibility to infections or autoimmune diseases. There is therefore great interest in the characterization of canine TLRs.

**Results:**

Polymorphisms in canine TLRs have been characterized by massive sequencing after enrichment of their exonic regions. DNAs from 335 dogs (seven different breeds) and 100 wolves (two different populations) were used in pools. The ratio of SNP discovery was 76.5% (in relation to CanFam 3.1); 155 out of 204 variants identified were new. Functional annotation identified 64 non-synonymous variants (43 new), 73 synonymous variants (56 new) and 67 modifier variants (57 new). 12 out of 64 non-synonymous variants are breed or wolf specific. TLR5 has been found to be the most polymorphic among canine TLRs. Finally, a TaqMan OpenArray® plate containing 64 SNPs with a possible functional effect in the protein (4 frameshifts and 60 non-synonymous codons) has been designed and validated.

**Conclusions:**

Non-synonymous genetic variation has been characterized in exonic regions of canine Toll-like Receptors. The TaqMan OpenArray® plate developed to capture the individual variability that affects protein function will allow high-throughput genotyping either to study association to infection susceptibility or even TLR evolution in the canine genome.

**Electronic supplementary material:**

The online version of this article (doi:10.1186/2052-6687-1-11) contains supplementary material, which is available to authorized users.

## Lay summary

Toll-like receptors (TLRs) are pattern recognition receptors (PRRs) and are the primary sensors of pathogens in the body. Genetic variants could be associated with differences in breed response to pathogens and also to susceptibility to infections and/or autoimmune diseases. There is great interest in the characterization of canine TLRs.

Genetic variation in canine TLRs has been characterized using massive parallel sequencing. DNA from 335 dogs (seven breeds: Beagle, German Shepherd dog, Yorkshire terrier, French bulldog, Boxer, Labrador and Shar Pei) plus 100 wolves (two populations: Iberian and Russian) were sequenced in 16 pools of 25 dogs or 50 wolves. In total, we found 204 variants, of which 155 were new. Comparison of these variants with the published dog genome sequence (called CanFam 3.1) Functional annotation identified 64 non-synonymous variants (43 new), 73 synonymous variants (56 new) and 67 modifier variants (57 new). Twelve of 64 non-synonymous variants were breed or wolf specific. TLR5 has been found to be the most polymorphic among canine TLRs. Finally, a TaqMan OpenArray(R) plate containing 64 SNPs with a possible functional effect in the protein (4 frameshifts and 60 non-synonymous codons) has been designed and validated.

Non-synonymous genetic variation has been characterized in exonic regions of canine Toll-like Receptors. The TaqMan OpenArray(R) plate developed to capture the individual variability that affects protein function will allow high-throughput genotyping either to study association to infection susceptibility or even TLR evolution in the canine genome.

## Background

Toll-like receptors (TLRs) are the most widely studied pattern recognition receptors (PRRs) and are considered to be the primary sensors of pathogens in innate immunity. These molecules are constituted by leucine-rich repeat (LRR) domains, a unique intramembrane domain and a Toll/Interleukin-1 receptor (TIR) domain. Pathogen-associated molecular Patterns (PAMPs) are sensed through LRR domain, and signals are transduced through TIR domain, which is always located in the cytoplasm, in order to activate innate immunity response (for a review, see [1]).

Ten TLRs have been identified in dogs. TLRs can be classified into groups, depending on the PAMPs detected and their cellular location. TLR 1, 2, 4, 5 and 6 detect pathogen extracellular components. TLRs 3, 7, 8 and 9 target nucleic acids. The ligand for TLR10 is unknown [2].

Another way to classify TLRs is their cellular location. TLRs 1, 5, 6 and 10 are expressed at the cell surface and mainly recognize bacterial products. On the other hand, TLRs 3, 7, 8 and 9 are located almost exclusively in intracellular compartments and are specialized in recognition of nucleic acids, with self versus non-self discrimination provided by the exclusive localization of the ligands rather than their different molecular structure from that of the host. TLRs 2 and 4 can be located both on the cell surface and intracellular [2, 3]. In this study, TLRs will be divided in two groups: TLRs 1, 2, 4, 5, 6 and 10 as extracellular TLRs and TLRs 3, 7, 8 and 9 as intracellular TLRs and nucleic acid sensors.

TLRs are conserved through evolution, from Drosophila to mammals (reviewed at [4]), because of its essential role in innate immunity. However, there are significant distinctions between intracellular and extracellular TLRs. Intracellular TLRs do not accept much variability, because they have evolved under strong purifying selection [5]. Viruses can only be detected through their nucleic acids; therefore intracellular TLRs have an essential non-redundant role in host survival. Moreover, mutations in those TLRs could end up with an autoimmune disease against own nucleic acids or with high susceptibility to some viral infections. On the other hand, membrane or extracellular TLRs have evolved under less evolutionary pressure, due to they can recognize one pathogen through different PAMPs (immunological redundancy). So they show a higher rate of damaging non-synonymous and STOP mutations.

Although infective pressure that has reached these molecules is one of the main mechanisms of evolution, it is not the only one. Non-adaptative evolution has also an important role, through genetic drift, bottlenecks and migratory routes [6]. This kind of evolution should be seen in dogs, due to a first bottleneck with domestication and a second one for the artificial selection of breeds [7]. For these reason it should be taken into account the need for dealing with different breeds, and even with the wolf, for the analysis of canine TLR polymorphism.

In humans, many studies are addressed to find out possible links between some TLR polymorphism and susceptibility or resistance to disease (for a review see [6]). Some genetic variants in TLRs in dogs could be associated to differences in breed innate immune response to pathogens and thus to susceptibility to infections or autoimmune diseases. So far, polymorphisms in TLR4 and TLR5 have been associated with Inflammatory Bowel disease (IBD) in German Shepherd dogs (GSD) [8], but only protective SNPs from TLR5 have been associated with IBD in other 38 dog breeds [9] There is therefore great interest in the characterization of canine TLRs. TLR5 risk-associated haplotype for canine IBD confers hyper-responsiveness to flagellin [10]. Moreover, dogs with spontaneous IBD exhibit alterations in the enteric microbiota, which bear resemblance to dysbiosis reported in humans with chronic intestinal inflammation [11].

Although no other polymorphisms have been associated to illness in dogs until date, some studies have reported differential expression of some TLRs related to inflammatory or infectious diseases, such as TLR2 in IBD [12], TLRs 2, 4, 5 and 9 in chronic enteropathies in German Shepherd [13, 14]; TLR4 in osteoarthritis [15] and in infected canine endometrium [16]; TLRs 1-4, 6-10 in sino-nasal aspergillosis and idiopathic lymphoplasmacytic rhinitis [17]; and TLR2 and TLR9 in *Leishmania* infected dogs [18, 19].

So our aim is the analysis of genetic variation in exonic regions of canine TLRs by massive sequencing, focusing in non-synonymous substitutions and their segregation in different dog breeds and wolf populations. A second objective is to design and validate a TaqMan OpenArray® plate of SNPs with a possible functional effect in the protein (STOP, frameshift and non-synonymous codons). High-throughput genotyping of canine TLRs with this TaqMan OpenArray® plate will allow studying the association of non-synonymous variants with individual differences in immune response, their relationship with either the commensal or the disease associated microbiota and TLR evolution in the canine genome.

## Results

We have identified 156 new variants in canine TLRs by massive sequencing after the enrichment of exonic regions. DNAs from 335 dogs (seven breeds) and 100 wolves (two populations) were pooled in 16 pools and sequenced in 2 lanes of Illumina Hiseq, with a mean coverage value of 15,162.23. Dog breeds included were Beagle, Labrador, German Shepherd, Yorkshire, French Bulldog, Boxer and Shar Pei. Wolves included were Iberian (*Canis lupus signatus*) and Russian (European grey wolf, *Canis lupus lupus*). A total of 204 variants were detected: 193 SNP and 11 insertions or deletions (1 to 18 bases). Only one of the indels (insertion/deletion) mapped to an exonic region (TLR7 3′ UTR), meanwhile the others were mapping to intronic regions (5 out of 11) and intergenic regions upstream or downstream a TLR gene (5 out of 11). The SNPs identified were classified by functional annotation from ENSEMBL [20] (effect and effect impact): 73 synonymous variants, 64 non-synonymous variants and 67 modifier variants which include intergenic (upstream and downstream a TLR gene), intronic and 3′ UTR (untranslated region) variants (see Table 1). None of the variants detected in the pools analyzed had a high effect (STOP codon, frameshift mutation or splicing) on the protein function. The ratio of SNP discovery was 76.5% (in relation to CanFam 3.1); 156 out of 204 variants identified were new: 43/64 non-synonymous variants (nsSNP), 56/73 synonymous variants (synSNP) and 57/67 modifier variants.

**Table 1:** **Variants detected in canine TLRs by massive sequencing**

		Extracellular TLRs						Intracellular TLRs				
Effect impact	SNP effect	TLR1	TLR2	TLR4	TLR5	TLR6	TLR10	TLR3	TLR7	TLR8	TLR9	Total
**Low**	Syn coding	2	5	4	28	2	5	6	6	8	7	73
**Moderate**	Non-syn coding	4	3	12	23	4	3	1	3	4	7	64
***Total coding SNP (cSNP)***		***6***	***8***	***16***	***51***	***6***	***8***	***7***	***9***	***12***	***14***	***137***
**Modifier**	Downstream	1	4	0	0	2	3	0	0	2	1	12
	Intron	0	0	6	3	0	0	10	7	0	2	28
	Upstream	0	6	0	0	2	1	1	1	1	0	12
	UTR 3′	0	0	4	0	0	0	0	10	0	0	14
***Total non coding SNP (ncSNP)***		***1***	***10***	***10***	***3***	***4***	***4***	***11***	***18***	***3***	***3***	***66***
***Total SNP***		***7***	***18***	***26***	***54***	***10***	***12***	***18***	***27***	***15***	***17***	***204***

Variants are classified according to their effect on the protein and their spread along cell surface or intracellular TLRs.

Genetic variation differs among all TLRs. Variants detected in either extracellular or intracellular canine TLRs by massive sequencing and its classification according their effect in the protein are shown in Table 1. TLR5 gene presents the highest polymorphism, with 28 synonymous changes and 23 non-synonymous changes (Additional file 1, Table 1), although it also codifies for the longest annotated protein (1422 aminoacids).

Table 2 shows the aminoacid (AA) change ratio, which are AA changes caused by nsSNPs or frameshift mutations divided by total number AA for each one of the TLRs. The AA change ratio confirms that indeed TLR5 and TLR4 are the most polymorphic ones. On the other hand, TLR3 seem to be the most conserved receptor, just presenting one AA change in 905 AA.

**Table 2:** **Total number of variants affecting protein in extracellular and intracellular TLRs**

Canine gene	ENSEMBL protein ID	Protein length (aa)	AA change ratio ^a^
**Extracellular TLRs**			
TLR1	ENSCAFP00000032660	790	1/113
TLR2	ENSCAFP00000012269	785	1/196
TLR4	ENSCAFP00000031395	833	1/69
TLR5	ENSCAFP00000016726	1422	1/53
TLR6	ENSCAFP00000023836	797	1/199
TLR10	ENSCAFP00000023840	807	1/269
**Intracellular TLRs**			
TLR3	ENSCAFP00000011004	905	1/905
TLR7	ENSCAFP00000017193	1121	1/374
TLR8	ENSCAFP00000031505	1038	1/260
TLR9	ENSCAFP00000030804	1032	1/129

Variants from CanFam 3.1 have been added to variants identified by massive sequencing in this table. ^a^AA change ratio: aminoacid changes caused by nsSNPs or frameshift mutations divided by the length of the protein in aminoacids.

### Non-synonymous SNPs

A more exhaustive analysis was performed for the 64 nsSNP detected through massive sequencing, because they are expected to have a greater effect on the protein function. First, a glimpse on allelic frequencies of the nsSNP was performed. The frequencies of the alternative allele for all 64 nsSNPs are shown for each breed and wolf pools in Additional file 2.

Allelic frequencies for alternative variants in nsSNPs differ among breeds. Beagle and Russian wolf are the most variable pools, with 35 out of 64 nsSNPs segregating. Some of the variants identified are breed-specific (8 out of 64) or wolf-specific (4 out of 64). Most of the breed specific variants are found in TLR5 and TLR4, which as seen before, are the most polymorphic TLRs. German Shepherd dogs (GSD) and wolf share 3 nsSNPs, all located in TLR4. The same happens with Shar Pei and wolf, they share 3 nsSNPs located in TLR2, TLR5 and TLR6.

SNPs with a MAF (Minor allele frequency) <0.05 have been considered to be fixed in the cohort [21]. Usually it is the reference allele the one which is fixed, but in some cases (perhaps due to bad annotation of the SNP) is the alternative one. Iberian wolves’ cohort is the one with more fixed variants, with only 24 out of 64 that are segregating, followed by Yorkshire and Boxer, with 25 out of 64 segregating variants.

### Predicted impact of canine TLRs amino acid substitutions

Polyphen-2 [22, 23], SIFT [24, 25], and PROVEAN [26, 27] tools were used in order to predict the effect of each nsSNP in the protein structure. Each of these tools uses a different algorithm to predict the consequence of the aminoacid change on the protein and classifies it as benign/tolerated/neutral or damaging/affect protein function/ deleterious (for more detail, see Methods). 28 out of 64 nsSNPs were predicted to have an effect on the protein structure by at least one of the tools used (Table 3). When frequency of the alternative variant was high for all the cohorts tested, the alternative allele was exchanged with the reference allele in ENSEMBL sequences [20] in order to perform the Polyphen-2 analysis with the less frequent variant as the “alternative variant”. Therefore, SNPs with frequencies greater than 0.25 for the alternative allele were tested also for the annotated reference allele. Then, 3 more SNPs were predicted to affect the protein structure (indicated as reference on the column dbSNP ID in Table 3). When considering also these ones, 31 out of 64 nsSNPs (48%) were predicted to have an impact on the protein structure. Results from Polyphen-2, SIFT and PROVEAN were convergent in predicting damaging effects for 8 out of 31 nsSNPs (27%). On the other hand, 6 out of 64 nsSNPs were not correctly predicted, giving unknown or low confidence results, because they were not aligning to enough similar sequences to give a reliable result. Curiously most of this nsSNPs were located on the N-terminal region of TLR5.

**Table 3:** **Non-synonymous SNPs predicted to impact protein function either by Polyphen-2, SIFT or PROVEAN**

Canine gene	Position	SNP	dbSNP ID	AA Subst	Protein domain ^a^	Polyphen-2 result	SIFT result	Provean result	Variant freq (dog) ^b^	Variant freq (wolf) ^b^
**EXTRACELLULAR TLRs**										
TLR1	3:73542337	G/T	rs23585044	S29I	ncp	Pos. damaging	Tolerated	Neutral	0,36	0,77
	3:73543092	T/G	new	S281A	ncp	Pos. damaging	Tolerated	Neutral	0,06	0,00
	3:73543825	C/T	new	A525V	LRRCT^2^	Pos. damaging	Tolerated	Deleterious	0	0,11
TLR2	15:51463020	C/A	rs22410121	S46Y	ncp	Pos. damaging	Tolerated	Neutral	0,10	0,00
	***15:51464430***	***C/T***	***new***	***S516L***	***ncp***	***Prob. damaging***	***Aff. function***	***Deleterious***	***0,14***	***0***
TLR3	16:44623632	C/G	new	E176D	ncp	Pos. damaging	Tolerated	Neutral	0,16	0,12
TLR4	11:71356420	C/T	reference^1^	A8V	ncp	Prob. damaging	Tolerated	Neutral	0,77	0,57
	11:71360887	G/A	new	V82M	ncp	Pos. damaging	Tolerated	Neutral	0,09	0,15
	***11:71364581***	***T/C***	***rs22145736***	***L167P***	***ncp***	***Prob. damaging***	***Aff. function***	***Deleterious***	***0,15***	***0***
	11:71364681	A/C	reference^1^	Q200H	ncp	Pos. damaging	Aff. function	Neutral	0,88	0,23
	11:71365810	A/G	new	T577A	LRRCT^3^	Pos. damaging	Aff. function	Neutral	0,01	0
TLR5	38:23702837	C/T	rs9070447	R269C	ncp	Prob. damaging	Aff. function	Neutral	0,19	0,01
	38:23702918	G/A	new	V296I	ncp	Pos. damaging	Tolerated	Neutral	0,05	0,26
	38:23703629	G/A	new	G533S	ncp	Prob. damaging	Tolerated	Neutral	0,02	0
	38:23704331	G/T	new	D767Y	ncp	Prob. damaging	Tolerated	Deleterious	0,04	0
	38:23704531	C/G	new	N833K	LRRCT	Pos. damaging	Tolerated	Deleterious	0	0,06
	38:23704562	C/T	new	R844C	LRRCT	Pos. damaging	Tolerated	Neutral	0,04	0,39
	***38:23704581***	***C/T***	***reference*** ^***1***^	***S850L***	***LRRCT***	***Prob. damaging***	***Aff. function***	***Deleterious***	***0,68***	***0,02***
	***38:23704695***	***T/G***	***new***	***F888C***	***low complexity***	***Prob. damaging***	***Aff. function***	***Deleterious***	***0,04***	***0***
	38:23705081	C/T	new	H1017Y	TIR	Benign	Aff. function	Neutral	0,02	0
	38:23705264	G/A	new	A1078T	TIR^4^	Pos. damaging	Aff. function	Neutral	0,07	0,00
TLR6	3:73521250	A/G	new	Y182C	ncp	Prob. damaging	Tolerated	Deleterious	0,01	0,09
	***3:73522074***	***C/T***	***new***	***L457F***	***ncp***	***Prob. damaging***	***Aff. function***	***Deleterious***	***0,03***	***0***
	3:73522242	G/A	rs23570247	D513N	ncp	Pos. damaging	Tolerated	Neutral	0,73	1,00
	***3:73522441***	***C/T***	***new***	***P579L***	***LRRCT***	***Pos. damaging***	***Aff. function***	***Deleterious***	***0,01***	***0,07***
TLR10	***3:73569402***	***C/T***	***rs23518574***	***T361M***	***ncp***	***Prob. damaging***	***Aff. function***	***Deleterious***	***0,13***	***0,12***
	3:73570681	T/A	new	F787L	TIR^5^	Pos. damaging	Low confidence	Neutral	0,00	0,39
**INTRACELLULAR TLRs**										
TLR8	***X:9397240***	***T/C***	***new***	***V157A***	***ncp***	***Pos. damaging***	***Aff. function***	***Deleterious***	***0,06***	***0***
TLR9	20:37544129	G/A	new	V87I	ncp	Benign	Aff. function	Neutral	0,02	0
	20:37546230	C/T	new	P787L	ncp	Pos. damaging	Tolerated	Neutral	0,22	0,24
	20:37546454	C/T	new	R862W	ncp	Prob. damaging	Tolerated	Neutral	0,2	0

In italics, SNPs that are predicted to have an effect on protein function by the three algorithms. ^a^ncp, no confident prediction. ^b^Observed frequency by massive sequencing. ^1^reference allele tested as the alternative in the SNP. ^2^Leucine Rich Repeat C-terminal (LRRCT) domain predicted from aminoacid 528 to 582. ^3^LRRCT domain predicted from aminoacid 579 to 629. ^4^TIR domain predicted from aminoacid 927 to 1074. ^5^TIR domain predicted from aminoacid 641 to 784.

Protein structure of the canine TLRs was assessed using SMART [28, 29], which predicts domains taking into account aminoacid sequences: 6 out of 31 nsSNPs predicted to be damaging in canine TLRs were found to be in a Leucine Rich Repeat C-terminal (LRRCT) or really close to it. Only 1 out of 31 was found to affect TIR domain in TLR 5, other 2 were found to be really close to this domain in TLR5 and 10. With the exception of these last ones, nsSNPs were in most cases located in the sensor domain of TLRs (Table 3).

**Figure 1:**
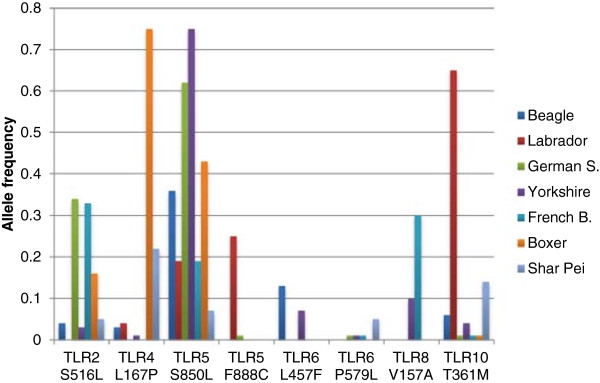
**Breed allelic frequencies for the 8 nsSNP with a damaging prediction from Polyphen-2, SIFT and PROVEAN.**

Frequencies in Table 3 are an average of all dog pools tested and both wolf populations respectively, so all variants are polymorphic (MAF > 0.05) at least in one breed. 17 out of 31 show a MAF > 0.05 when considering the average frequencies in all the pools together (15 out of 31 with MAF > 0.05 in wolf populations). However, as mentioned above, frequencies of nsSNPs differ among breeds (see Additional file 2). It’s worthy to note the differences on the alternative allele frequency observed for the 8 nsSNPs that were predicted to affect protein function by the three tools used (Figure 1).

### TaqMan open array design and SNP validation

A TaqMan OpenArray® plate has been developed for the validation of the nsSNPs by individual genotyping (Table 4). This panel contains (i) 27 out of 31 nsSNPs that were predicted to have an impact on the protein structure (4 wolf-specific SNPs were not considered for the array: TLR1 A525V, TLR5 N833K, TLR6 P579L and TLR10 F787L; see Table 3); (ii) 28 out of the 33 remaining nsSNPs segregating in dogs (5 SNPs that were not suitable for a correct primer design were rejected for posterior analysis: TLR4 T36A, TLR4 T36I, TLR5 T243A, TLR5 Q213R and TLR9 A442V); and (iii) 4 frameshift and 4 non-synonymous TLR polymorphisms described on CanFam 3.1 but not detected in our cohorts (see Table 4). One of the non-synonymous variants added (rs23572381, TLR1 N634K) was designed with two different TaqMan assays due to the presence of other variants close to the interrogated SNP.

**Table 4:** **Non-synonymous SNPs and frameshift mutations of canine TLRs in the TaqMan Open Array plate**

Canine gene	SNP	Chr:bp position	dbSNP ID	AA Subst	Previous detected ^a^	Validated?
TLR1	G/T	3:73542337	rs23585044	S29I	Massive seq	YES
	T/G	3:73543092	new	S281A	Massive seq	YES
	G/A	3:73543185	new	V312I	Massive seq	YES
	T/A	3:73544153	rs23572381	N634K^1^	CanFam 3.1	YES^2^
	T/A	3:73544153	rs23572381	N634K^1^	CanFam 3.1	NO
	G/A	3:73544221	rs23572380	S657N	CanFam 3.1	YES^2^
TLR2	C/A	15:51463020	rs22410121	S46Y	Massive seq	YES
	A/0	15:51464076	rs8958543	A398-	CanFam 3.1	YES^2^
	C/T	15:51464430	new	S516L	Massive seq	YES
	C/T	15:51464700	new	T606M	Massive seq	YES
TLR3	C/G	16:44623632	new	E176D	Massive seq	YES
TLR4	T/C	11:71356420	rs22120766	V8A	Massive seq	NO
	G/C	11:71360743	rs22157966	A34P	Massive seq	YES
	G/A	11:71360887	new	V82M	Massive seq	YES
	T/C	11:71364581	rs22145736	L167P	Massive seq	YES
	C/A	11:71364681	rs22189454	H200Q	Massive seq	YES
	A/G	11:71364769	rs22189456	K230E	Massive seq	YES
	G/A	11:71365120	new	A347T	Massive seq	YES
	A/T	11:71365652	rs22124023	E524V	Massive seq	YES
	A/G	11:71365810	new	T577A	Massive seq	YES
	G/A	11:71365888	rs22123995	E603K	Massive seq	YES
TLR5	G/A	38:23702193	rs24029590	G54E	CanFam 3.1	NO
	0/C	38:23702251	rs9070448	-74C	CanFam 3.1	YES^2^
	A/G	38:23702514	rs9070450	Y161C	CanFam 3.1	NO
	A/C	38:23702539	new	E169D	Massive seq	YES
	G/A	38:23702562	new	S177N	Massive seq	YES
	G/C	38:23702640	rs9070451	R203P	Massive seq	YES
	T/C	38:23702684	rs9070452	W218R	Massive seq	NO
	C/T	38:23702837	rs9070447	R269C	Massive seq	YES
	G/A	38:23702918	new	V296I	Massive seq	YES
	T/C	38:23703180	new	L383S	Massive seq	YES
	G/A	38:23703237	new	R402Q	Massive seq	YES
	G/A	38:23703279	new	R416Q	Massive seq	YES
	T/0	38:23703591	rs9125247	T520-	CanFam 3.1	YES^2^
	G/A	38:23703629	new	G533S	Massive seq	YES
	G/A	38:23704233	new	R734Q	Massive seq	YES
	G/T	38:23704331	new	D767Y	Massive seq	YES
	C/T	38:23704562	new	R844C	Massive seq	YES
	T/C	38:23704581	rs24029975	L850S	Massive seq	YES
	T/G	38:23704695	new	F888C	Massive seq	YES
	G/A	38:23704718	new	A896T	Massive seq	YES
	C/T	38:23705081	new	H1017Y	Massive seq	YES
	G/A	38:23705090	new	G1020S	Massive seq	YES
	G/A	38:23705178	new	R1049Q	Massive seq	YES
	G/A	38:23705264	new	A1078T	Massive seq	YES
TLR6	A/G	3:73521250	new	Y182C	Massive seq	YES
	C/T	3:73522074	new	L457F	Massive seq	YES
	G/A	3:73522242	rs23570247	D513N	Massive seq	YES
TLR7	C/G	X:9334108	new	A16G	Massive seq	YES^2^
	C/A	X:9355727	new	F167L	Massive seq	YES
	C/T	X:9358423	new	P1066L	Massive seq	YES
TLR8	T/C	X:9397240	new	V157A	Massive seq	YES
	G/A	X:9397663	new	R298Q	Massive seq	YES
	G/A	X:9398094	rs24607342	G442S	Massive seq	YES
	G/A	X:9398827	rs24607358	R686H	Massive seq	YES
TLR9	G/A	20:37544129	new	V87I	Massive seq	YES
	0/A	20:37544851	rs9188882	-328A	CanFam 3.1	YES^2^
	A/G	20:37545011	new	K381E	Massive seq	YES
	C/A	20:37545245	new	P459T	Massive seq	YES
	A/G	20:37546031	rs22882109	S721G	Massive seq	YES
	C/T	20:37546230	new	P787L	Massive seq	ND^**3**^
	C/T	20:37546454	new	R862W	Massive seq	YES
TLR10	C/T	3:73569402	rs23518574	T361M	Massive seq	YES
	A/G	3:73570094	new	M592V	Massive seq	YES

^a^Massive seq indicates a SNP variant detected in our cohorts. An “rs” name is indicated in dbSNP ID if the SNP is annotated in CanFam 3.1. ^1^SNP considered twice with a different surrender SNP in order to detect it. ^2^Assay has been validated technically, although not genetically because all individuals have only the reference allele. ^3^ND (not determined), there are incongruent results: massive sequencing showed that this SNP was present at a frequency of 0.2 in all breeds tested, whereas it has not been genotyped through TaqMan OA plate.

A total of 99 DNA samples of the first massive sequencing pools were chosen to be individually genotyped in order to validate the SNPs with the TaqMan Open Array® designed: 15 Beagle, 15 Boxer, 14 French bulldog, 15 Labrador, 15 German Shepherd dog, 13 Yorkshire and 12 Shar Pei were used. One Shar-Pei and 2 Yorkshires do not pass the quality control for samples (call rate > 0.9) and were removed from the posterior analysis. Finally, analysis was performed with a total of 96 individuals. Fifty-nine out of the 64 SNPs (92%) included in the OpenArray have been successfully validated and all of them had a call rate greater than 0.9.

Some downstream analyses have been performed with the individual genotypes. However, it should be taken into account that these are just preliminary results, which need to be validated with larger cohorts of dogs.

All the TLR SNPs were in Hardy-Weinberg Equilibrium (HWE) on Beagle, Boxer, German Shepherd, Labrador and Shar-Pei. In Yorkshire, TLR10 has two SNPs in linkage disequilibrium which are not in HWE, one of them is predicted to affect protein function by the algorithms tested (Table 3). French Bulldog was the breed that had more SNPs that did not follow HWE proportions, with 3 SNPs in TLR4 and one SNP in TLR5 (Table 5). TLR7 and 8 were not included because they are both located in chromosome X.

**Figure 2:**
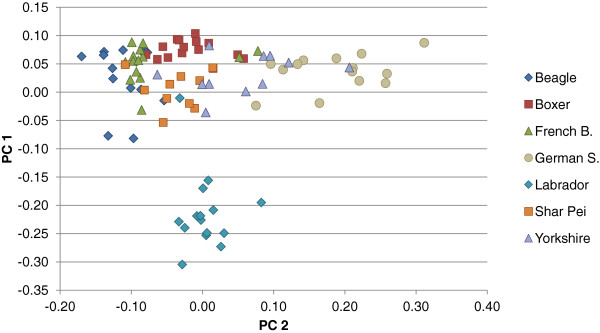
**Principal Component Analysis (PCA) plot of the two first components for canine TLRs.**

Principal components analysis (PCA) combined data from the individual genotypes obtained for the subset of SNPs which were not in linkage disequilibrium. It was used to illustrate if dogs cluster by breed for genetic variants in TLRs. The first two components from the PCA have been plotted in Figure 2. Visual examination of this plot shows overlapping for most breeds, excluding Labrador and perhaps German Shepherd, which seem to be more differentiated for these receptors.

**Table 5:** **SNPs in different breeds that are not in Hardy-Weinberg Equilibrium (p < 0.05)**

Breed	Canine gene	AA change	SNP	Genotypes ^a^	p-value	SNP prediction ^b^
Yorkshire	TLR10	T361M	C/T	1/1/9	0.0416978	Prob. damaging
Yorkshire	TLR10	M592V	A/G	1/1/9	0.0416978	Benign
French B.	TLR4	V82M	G/A	4/2/8	0.0099493	Pos. damaging
French B.	TLR4	H200Q	C/A	6/1/7	0.0013535	Pos. damaging*
French B.	TLR4	K230E	A/G	6/1/7	0.0013535	Benign
French B.	TLR5	S177N	G/A	0/11/3	0.0154748	Benign

^a^genotypes, indicate genotype count for reference homozygotes, heterozygotes and alternative homozygotes. ^b^SNP prediction, using Polyphen-2 classification. *possibly damaging when reference allele is tested as alternative in the SNP.

## Discussion

Canine breed specific variants in TLRs could be associated to differences in breed innate immune response to pathogens and thus to susceptibility to infections or autoimmune diseases. So far, polymorphisms in TLR4 and TLR5 have been associated with IBD in German Shepherd dogs [8], but only protective SNPs from TLR5 have been associated with IBD in other 38 dog breeds [9]. There is therefore great interest in the characterization of canine TLRs. Different dog breeds and 2 different populations of wolves (Iberian and Russian) were included in the analysis to represent some of the major phylogenetic radiations: Wolves, Ancient&Spitz breeds, Scent hounds, Working dogs, Mastiff-like dogs, Small Terriers and Retrievers [30]. A total of 204 variants have been discovered and functionally annotated in exonic regions of canine TLRs by massive sequencing: 155 of the variants were new in relation to the most recent annotation of the canine genome (CanFam 3.1; September 2012). Variants have been functionally annotated and correspond to 64 non-synonymous variants (43 new), 73 synonymous variants (56 new) and 67 modifier variants (57 new). None of the variants detected in the pools analyzed had a high effect (STOP codon, frameshift mutation or splicing) on the protein function, although 4 frameshift mutations are annotated in CanFam 3.1.

SNPs functionally annotated as non-synonymous are expected to have a greater effect on protein function, and therefore a more exhaustive analysis was performed on them. Although allelic frequencies for nsSNPs differ among breeds and 12.5% of them are breed-specific (6.25% are wolf specific), dogs from different breeds share most non-synonymous variants in TLRs.

A TaqMan OpenArray® plate containing 64 SNPs with a possible functional effect in the protein (4 frameshifts and 60 nsSNPs) has been designed and validated. 55 out of 64 SNPs contained in the OpenArray® plate have been identified in this work through massive sequencing by HISEQ; the remaining 9 were obtained from CanFam 3.1.

As shown in Figure 2, the individual genotypes are not clustering by breed, with the exception of Labrador and German Shepherd dogs.

The functional impact of non-synonymous variants in dog TLRs was predicted using Polyphen-2, SIFT and PROVEAN. Knowing that TLRs are highly conserved receptors, it is not unexpected that half non-synonymous mutations in dogs have a benign effect, which agrees with results from similar approaches in other non-primate species such as bovine [31]. In dogs, TLR5 is the one that presents more damaging non-synonymous mutations (possibly damaging + probably damaging), followed by TLR4, both of them extracellular receptors.

Results from SIFT and Polyphen-2 from some nsSNPs located in TLR5 returned no output and no prediction (“unknown” or “low confidence”). In dogs, TLR5 was described as a longer protein compared to their homologs in other species. In CanFam 3.1 TLR5 has 1422 aminoacids, however other species like human, cow and pig have 858 aa, 858 aa and 856 aa, respectively. A protein BLAST was performed with the extra 5′ and 3′ TLR5 fragments, but no result was obtained. Furthermore, the 5′ sequence begins with ATG codon in the same phase as the initial coding ATG in other species, whereas the 3′ sequence eliminates the STOP codon due to some repeats in tandem (data not shown). So, bad annotation of this gene in CanFam3.1 is suggested. However, SNPs have been found in this region. In fact, there are 2 SNPs that had already been wrongly described as an aminoacid change (in ENSEMBL) moreover in this study 5 more SNPs have been detected. So it would be interesting to either determine the existence and functionality of these extra fragments in canine TLR5 cDNA or correctly annotate it in CanFam 3.1.

Intracellular TLRs, which detect nucleic acids, have less nsSNPs (15), moreover these are predicted to be less damaging variants than those identified in extracellular TLRs, suggesting that intracellular TLRs are selectively constrained. TLR9 is the intracellular TLR that accepts more nsSNPs in dogs, but the predicted effect of these nsSNPs is usually benign.

These results agree with previously reported data revealing major differences in the intensity of selection acting upon the different members of the TLR family. Different TLRs differ in their immunological redundancy, reflecting their distinct contributions to host defense [5, 32]. Intracellular TLRs act as nucleic acid sensors and have evolved under strong purifying selection, indicating their essential non-redundant role in host survival. Higher rates of damaging non-synonymous and nonsense mutations are tolerated in cell-surface or extracellular TLRs, which recognize compounds other than nucleic acids, suggesting a higher redundancy.

Location of the SNPs in the protein was approached using the software SMART [28], which identifies TLR domains using the aminoacid sequence. The intracellular TIR domain is highly conserved between different TLRs and species due to its involvement in intracellular signaling [33]. Also in dogs, TIR domains have few SNPs; only one is present in the predicted TIR domain (TLR5 H1017Y) and another two are really close to it (TLR5 A1078T and TLR10 F787L). Extracellular domains of TLRs are those that recognize PAMPs, and they have an enhanced susceptibility to mutate adapting to different microbiologic environments [33]. It can also be seen that a high number of mutations (some with damaging effects) are located in LRR domains, which form the extracellular domain of TLRs.

So far, polymorphisms in TLRs have been associated with Inflammatory Bowel disease (IBD) in German Shepherd dogs (GSD) and in other breeds. Variants in TLR5 previously reported to be associated to IBD (*G22A, C100T and T1844C* from [8, 9]) have been also detected in our cohorts and correspond with TLR5 T243A, TLR5 R269C and TLR5 L850S respectively.

**Figure 3:**
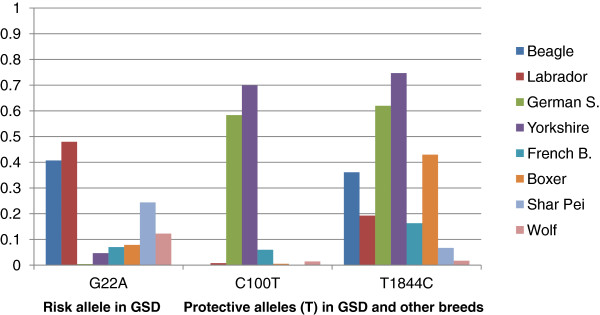
**Observed allele frequency of the alleles related with IBD in our pools (A in G22A, and T in both C100T and T1844C).**

SNP *G22A*, where the risk allele is A in *G22A* (corresponding to Thr in TLR5 T243A as named in this work), is found to be an additive allele. So when a GSD is homozygous for the risk allele it has more susceptibility to suffer IBD. This risk allele is not segregating in our GSD cohort. This could be due to the difference in the geographical origin of the GSD cohort between both studies. In [9] GSD are from UK, whilst our cohort is from Spain. SNPs *C100T* and *T1844C* were found to be significantly protective against canine IBD in many breeds [9]. The frequencies of the protective alleles (T in *C100T* and T in *T1844C* or Cys in TLR5 R269C and Leu in TLR5 L850S as named in this work) differ among breeds (Figure 3), with a frequency higher than 0.5 in Yorkshire and GSD.

## Conclusions

Polymorphisms in the exonic regions of canine TLRs have been characterized by massive sequencing and 156 out of 204 variants identified were new: 43/64 non-synonymous variants, 56/73 synonymous variants and 57/67 modifier variants. None of the variants detected in the pools analyzed had a high effect (STOP codon, frameshift mutation or splicing) on the protein function.

A TaqMan OpenArray® plate containing 64 SNPs with a possible functional effect in the protein (4 frameshifts and 60 nsSNPs) has been designed and validated to allow the high throughput genotyping of canine TLRs.

## Methods

### Ethics statement

The dogs in the study were examined during routinary veterinary procedures by the veterinary clinics participating in the study. All samples were collected for routine diagnostic and clinical purposes. The samples were obtained during veterinary procedures that would have been carried out anyway and DNA was extracted from residual surplus of samples and used in the study with verbal owner consent. This is a very special situation in veterinary medicine. As the data are from client-owned dogs that underwent normal veterinary exams, there was no “animal experiment” according to the legal definitions in Spain and the United Kingdom, and approval by an ethical committee was not necessary.

### DNA sources

Samples available from the DNA bank at the SVGM (Molecular Genetics Veterinary Service, UAB) were used. Total DNA from blood cells had been extracted either as described elsewhere [34] or using QIAamp DNA Mini Kit (Qiagen).

DNAs from 7 different dog breeds, including 50 Beagle, 50 German Shepherd, 50 Yorkshire, 35 French bulldog, 75 Boxer, 50 Labrador and 25 Shar Pei were used. All the dogs included in this study are from Spain region, and come from hospital population or normal pet dogs. Also 2 different populations of wolves, with 50 Iberian (*Canis lupus signatus*) and 50 Russian (European grey wolves, *Canis lupus lupus*), were analyzed. DNA pools were prepared with 200 ng of DNA from 25 unrelated dogs (with the exception of one pool of French bulldogs, with only 10 dogs). Two pools of each breed were analyzed, in exception of Shar Pei (only 1 pool) and Boxer (3 pools). Pools of wolves were of 50 individuals.

Some DNA samples of the first massive sequencing analyses were chosen to be individually genotyped in order to validate SNPs in the TaqMan Open Array® designed (15 Beagle, 15 Boxer, 14 French bulldog, 15 Labrador, 15 German Shepherd, 13 Yorkshire and 12 Shar Pei).

### Exon capture and massive sequencing for SNP discovery

Twenty exonic regions of 10 canine TLR genes annotated in CanFam 2.0 were chosen to perform the enrichment (see Additional file 3 with corresponding coordinates in CanFam 3.1).

Oligonucleotides were first automatically designed for the enrichment of selected regions [35]. Regions rejected in the automated design, because of the presence of gaps, repeats or shorter sizes than required (at least 120 nucleotides) were manually redesigned. Finally 235 ultra-long 120-mer biotinylated cRNA baits were designed to capture the exonic regions of canine TLRs (28,200 bases) by the Agilent Sure Select technique. High-throughput sequencing was performed using 2 lanes of Illumina HISEQ, with 8-labelled pools each, at CNAG (Centre Nacional d’Anàlisi Genòmica, Barcelona, Spain).

Sequences obtained were mapped to CanFam 3.1 (released September 2012). All pools were analysed together for variant calling, for better comparison. Alternative variant frequencies were estimated for each breed pool and wolf populations. The variants were annotated with statistical information from the Genome Analysis Tool Kit (GATK) and functional annotations were added from Ensembl using snpEff [36].

### Prediction of functional impact of non-synonymous SNPs

The functional impact of non-synonymous mutations detected was predicted using Polyphen-2 [22, 23], SIFT [25, 24] and PROVEAN [27, 26]. When the mean frequency of an alternative variant on the dog population analyzed was more than 0.25, both alleles of those SNPs were tested with algorithms mentioned before as reference and alternative.

Polyphen-2 classifies mutations in three categories: *benign*, *possibly damaging* and *probably damaging*. Polyphen algorithm considers protein structure and/or sequence conservation information for each gene [23]. SIFT is based on the evolutionary conservation of the amino acids within protein families performing multiple sequencing analyses using PSI-Blast algorithm. Highly conserved positions tend to be intolerant to substitution, whereas those with a low degree of conservation tolerate most substitutions. Therefore, it classifies each non-synonymous polymorphism as *tolerated* or *affect protein function* and provides also a confidence measure [24]. PROVEAN introduced a region-based “delta alignment score” which measures the impact of an amino acid variation not only based on the amino acid residue at the position of interest but also the quality of sequence alignment derived from the neighborhood flanking sequences. It classifies variants either as neutral or deleterious [26].

SMART was used in order to identify protein domains of each TLR using their aminoacid sequence [28].

### TaqMan OpenArray® design

A TaqMan OpenArray® was designed for genotyping and validating 64 SNPs with a possible functional effect in the protein. Selected SNPs and their surrounding sequences, 60 nucleotides upstream and 60 nucleotides downstream were introduced in Custom TaqMan® Assay Design Tool web site [37] from Life Technologies® to validate if the sequences were suitable for TaqMan assay design. Other SNPs in the context sequences were indicated with an “N” before the assays design. SNPs included are listed in Table 4.

Analysis was performed with the TaqMan Genotyper software v.1.3 (Applied Biosystems). Further analysis of individual genotypes was performed with SVS (version 7) of Golden Helix Inc. SNPs or samples that do not pass call rate >0.9 were removed for posterior analysis.

## Electronic supplementary material

Additional file 1: **Total number of detected synonymous and non-synonymous SNPs for each canine Toll-like receptor.** (DOCX 38 KB)

Additional file 2: **Frequencies of coding variants obtained by massive sequencing.** Frequency per breed (50 individuals approximately) and mean frequency per dog and wolf species. Frequency of the SNP is represented as the frequency of the alternative allele. (XLSX 37 KB)

Additional file 3: **Coordinates of exonic regions of 10 canine TLR genes as annotated in CanFam 3.1.** (DOCX 39 KB)
