# Supplementary figures and images for: Non-synonymous genetic variation in exonic regions of canine Toll-like receptors
Source: Canine Genet Epidemiol. 2014 Oct 22;1:11. doi: 10.1186/2052-6687-1-11 (PMC4579382; doi:10.1186/2052-6687-1-11)

**Additional file 1.**


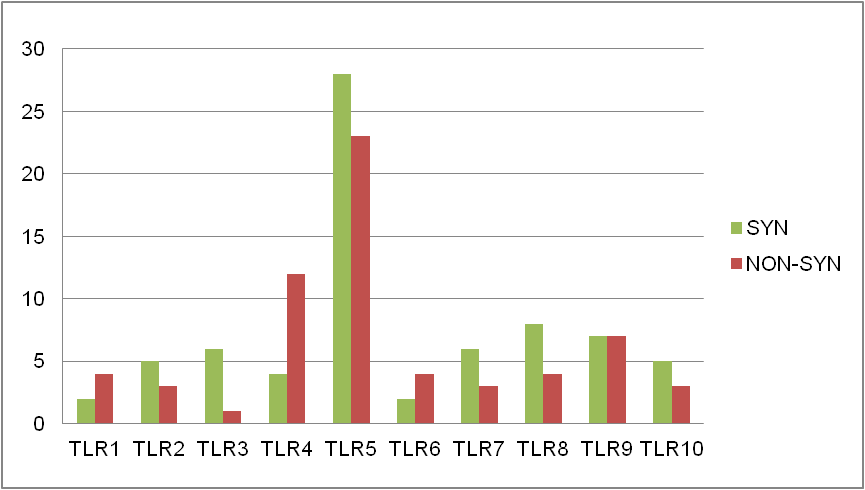

Supplement: Supplementary file 1 — Additional file 1: Total number of detected synonymous and non-synonymous SNPs for each canine Toll-like receptor. (DOCX 38 KB) [file 40575_2014_11_MOESM1_ESM.docx]
