# Supplementary material for: Non-synonymous genetic variation in exonic regions of canine Toll-like receptors
Source: Canine Genet Epidemiol. 2014 Oct 22;1:11. doi: 10.1186/2052-6687-1-11 (PMC4579382; doi:10.1186/2052-6687-1-11)
Supplement: Supplementary file 3 — Additional file 3: Coordinates of exonic regions of 10 canine TLR genes as annotated in CanFam 3.1. (DOCX 39 KB) [file 40575_2014_11_MOESM3_ESM.docx]

Additional file 3.

| **Gene** | **ENSEMBL ID** | **Chr** | **From** | **To** | **Length (bp)** |
| --- | --- | --- | --- | --- | --- |
| TLR1 | ENSCAFG00000024010 | chr 3 | 73542252 | 73544624 | 2372 |
| TLR2 | ENSCAFG00000008351 | chr 15 | 51462884 | 51465241 | 2357 |
| TLR3 | ENSCAFG00000007406 | chr 16 | 44625526 | 44625969 | 443 |
| TLR3 | ENSCAFG00000007406 | chr 16 | 44623524 | 44623715 | 191 |
| TLR3 | ENSCAFG00000007406 | chr 16 | 44619227 | 44621079 | 1852 |
| TLR3 | ENSCAFG00000007406 | chr 16 | 44618776 | 44619007 | 231 |
| TLR4 | ENSCAFG00000003518 | chr 11 | 71356398 | 71356490 | 92 |
| TLR4 | ENSCAFG00000003518 | chr 11 | 71360737 | 71360903 | 166 |
| TLR4 | ENSCAFG00000003518 | chr 11 | 71364342 | 71366571 | 2229 |
| TLR4 | ENSCAFG00000003518 | chr 11 | 71366774 | 71366809 | 35 |
| TLR5 | ENSCAFG00000011368 | chr 38 | 23702738 | 23705314 | 2576 |
| TLR6 | ENSCAFG00000016172 | chr 3 | 73520549 | 73520578 | 29 |
| TLR6 | ENSCAFG00000016172 | chr 3 | 73520703 | 73523090 | 2387 |
| TLR7 | ENSCAFG00000011698 | chr X | 9334187 | 9334277 | 90 |
| TLR7 | ENSCAFG00000011698 | chr X | 9355443 | 9359594 | 4151 |
| TLR8 | ENSCAFG00000023498 | chr X | 9396765 | 9399884 | 3119 |
| TLR9 | ENSCAFG00000023201 | chr 20 | 37543871 | 37545064 | 1193 |
| TLR9 | ENSCAFG00000023201 | chr 20 | 37545122 | 37546436 | 1314 |
| TLR9 | ENSCAFG00000023201 | chr 20 | 37546461 | 37546969 | 508 |
| TLR10 | ENSCAFG00000016175 | chr 3 | 73568321 | 73570744 | 2423 |
